# A multi-spectral myelin annotation tool for machine learning based myelin quantification

**DOI:** 10.12688/f1000research.27139.1

**Published:** 2020-12-21

**Authors:** Abdulkerim Çapar, Sibel Çimen, Zeynep Aladağ, Dursun Ali Ekinci, Umut Engin Ayten, Bilal Ersen Kerman, Behçet Uğur Töreyin

**Affiliations:** 1 Informatics Institute, Istanbul Technical University, Istanbul, 34469, Turkey; 2 Argenit Akıllı Bilgi Teknolojileri, Istanbul, 34469, Turkey; 3 Department of Electronics and Communication Engineering, Yildiz Technical University, Istanbul, 34220, Turkey; 4 Regenerative and Restorative Medicine Research Center, Istanbul Medipol University, Istanbul, 34810, Turkey; 5 School of Medicine Department of Histology and Embryology, Istanbul Medipol University, Istanbul, 34810, Turkey

**Keywords:** myelin annotation tool, myelin quantification, fluorescence images, machine learning, image analysis

## Abstract

Myelin is an essential component of the nervous system and myelin damage causes demyelination diseases. Myelin is a sheet of oligodendrocyte membrane wrapped around the neuronal axon. In the fluorescent images, experts manually identify myelin by co-localization of oligodendrocyte and axonal membranes that fit certain shape and size criteria. Because myelin wriggles along x-y-z axes, machine learning is ideal for its segmentation. However, machine-learning methods, especially convolutional neural networks (CNNs), requiring a high number of annotated images necessitates expert labor. To facilitate myelin annotation, we developed a workflow and a software for myelin ground truth extraction from multi-spectral fluorescent images. Additionally, we shared a set of myelin ground truths annotated using this workflow.

## Introduction

Myelin degeneration causes neurodegenerative disorders, such as multiple sclerosis (MS) ^1, 2^. There are no remyelinating drugs. Myelin quantification is essential for drug discovery, which often involves screening thousands of compounds ^3^. Currently, myelin quantification is manual, and labor-intensive. Automation of quantification using machine learning can facilitate drug discovery by reducing time and labor costs. However, myelin annotation suffers the same limitations as manual quantification. To assist researchers and bioimage analysts, we developed a workflow and a software for myelin ground truth extraction from multi-spectral fluorescent images.

**Figure 1.**
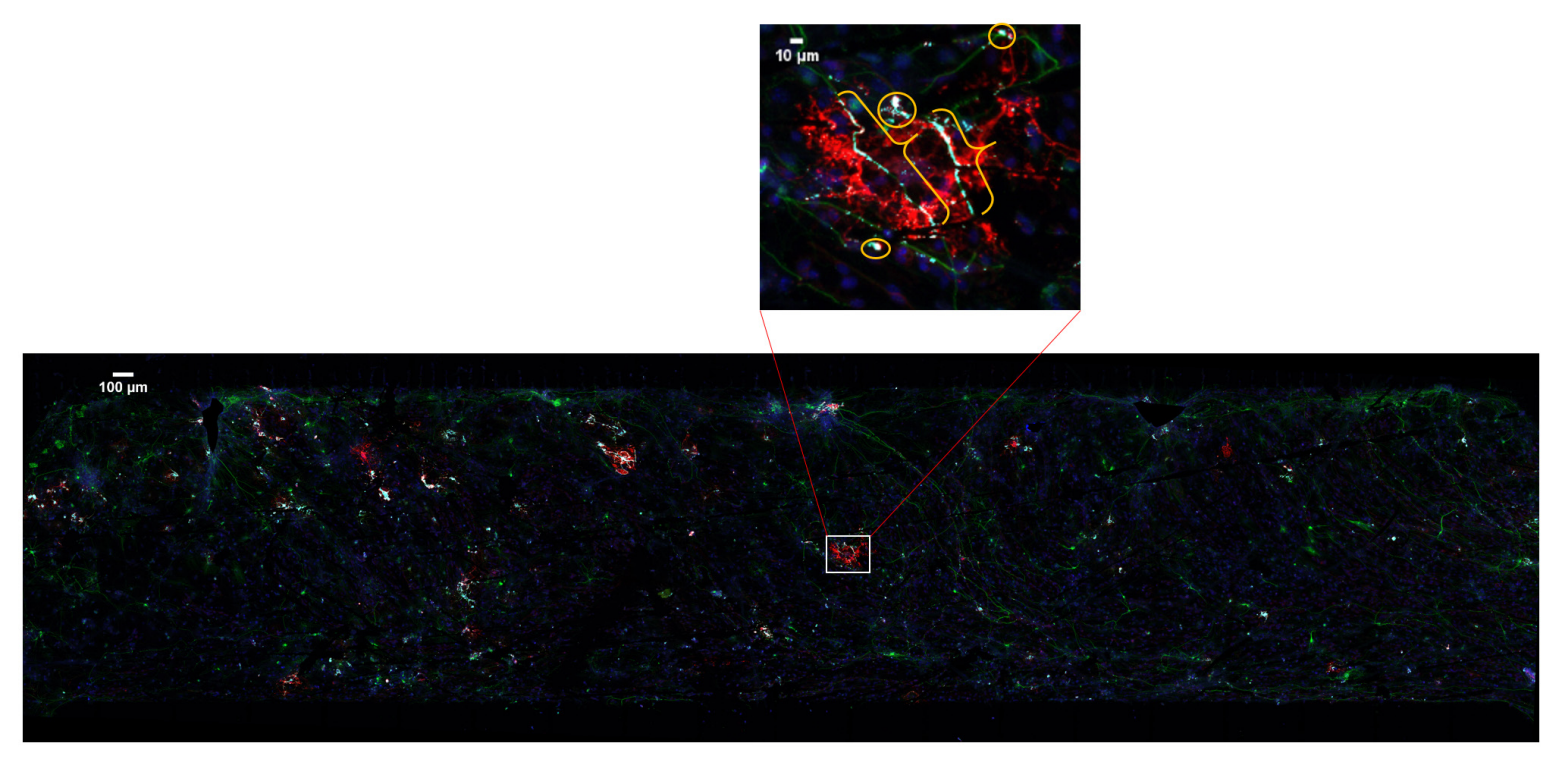
An example of multi-spectral fluorescent image. 20× confocal microscopy image tiles were stitched together covering approximately 2 × 8 mm by 30–50 μm volume. Boxed area is enlarged to show myelin (brackets) and the false positive pixels (circles).

Myelin is formed by oligodendrocytes wrapping the axons ^4^. It is identified by continuous co-localization of cellular extensions that span multiple channels and z-sections ( Figure 1). In our workflow, co-localizing pixels, candidate myelins, were determined using Computer-assisted Evaluation of Myelin (CEM) software that we previously developed ^5^. In the current study, the 3D Myelin Marking (CEM3D) tool ^6^ was developed to efficiently evaluate these candidate myelins and to extract myelin ground truths. Using CEM3D, an RGB-composite z-section image, corresponding CEM output image, and expert’s markings can be visualized simultaneously to decide whether to keep or remove candidate pixels (see *Implementation*). The user can move along x-y-z axes and show/hide channels, images and markings. Markings from the -1/+1 z-sections can be viewed simultaneously. Finally, CEM3D allows simultaneous visualization of myelin markings of two experts, which is important for inter-expert comparison.

Using the described workflow, we annotated five images encompassing approximately 2 × 8 mm by 30–50 μm volume. The entire process, which would have taken several weeks, took approximately 5 days. More than 30,000 feature images were extracted from these five images and were used for testing various machine-learning methods ^7– 9^. The annotated images, which are available with the manuscript, are a resource for the researchers working not only on myelin detection but also on segmenting multi-spectral images.

## Methods

### Image acquisition

Images were previously acquired ^5^. Briefly, co-cultures of mouse embryonic stem cell-derived oligodendrocytes and neurons were grown in microfluidic chambers. After myelin formation, cells were fixed in paraformaldehyde and were stained with 1:1,000 mouse or rabbit anti-TUJ1 (Covance), 1:50 rat anti-MBP (Serotec) and DAPI (Sigma). Images were acquired on Zeiss LSM 710 or 780 confocal microscopes as 10% overlapping tiles encompassing the entire myelination chamber. The z-axis, 30–50 µm, was covered by 1-µm-thick optical z-sections. The tiles were stitched together on Zen software (Zeiss). These images are available from the Image Data Resource ^10^.

### Implementation

**Figure 2.**
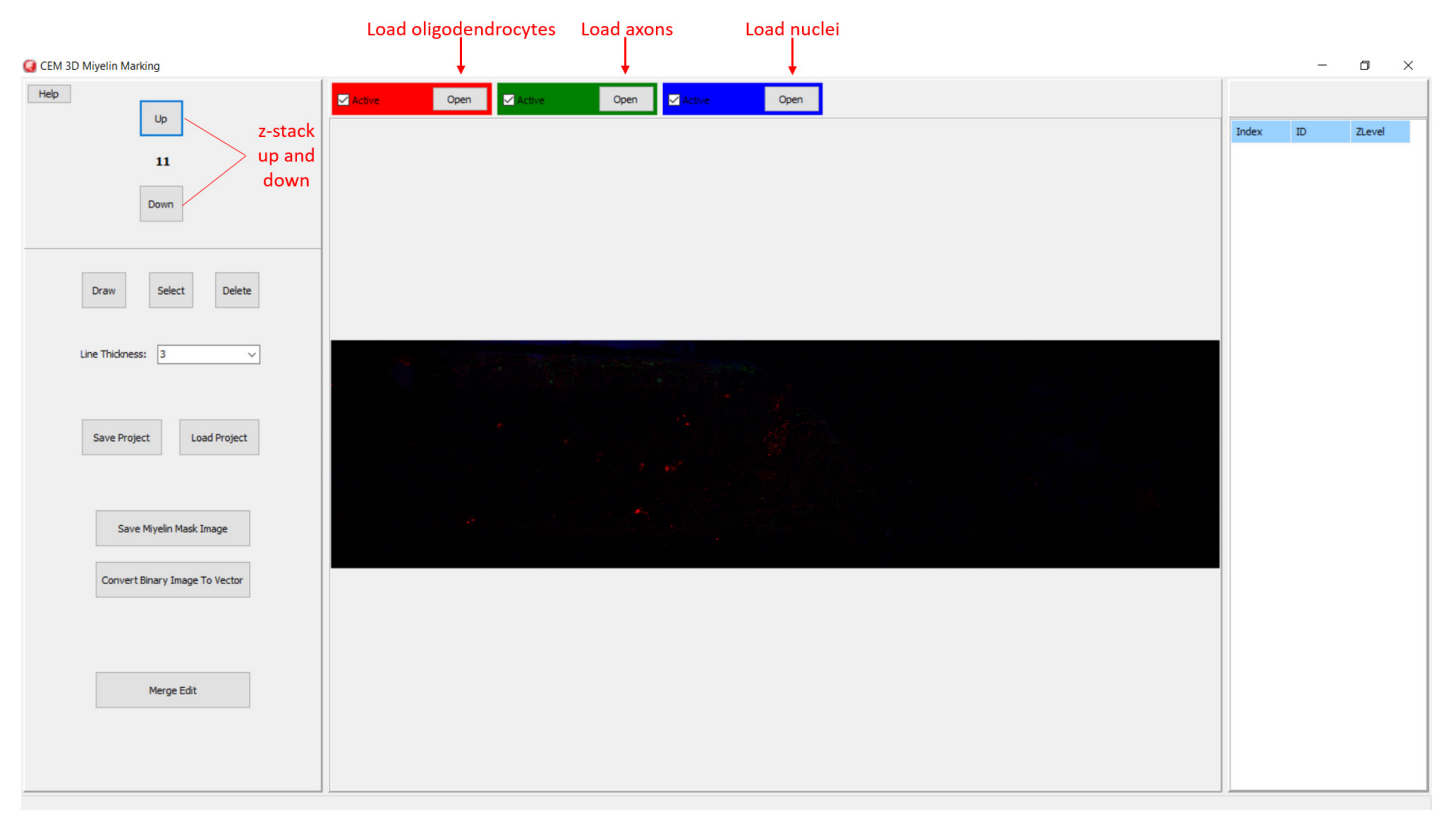
Starting a new project in CEM3D. Buttons for loading oligodendrocyte, axon, and nucleus images, and navigating the z-stack button to up and down are marked.

**Figure 3.**
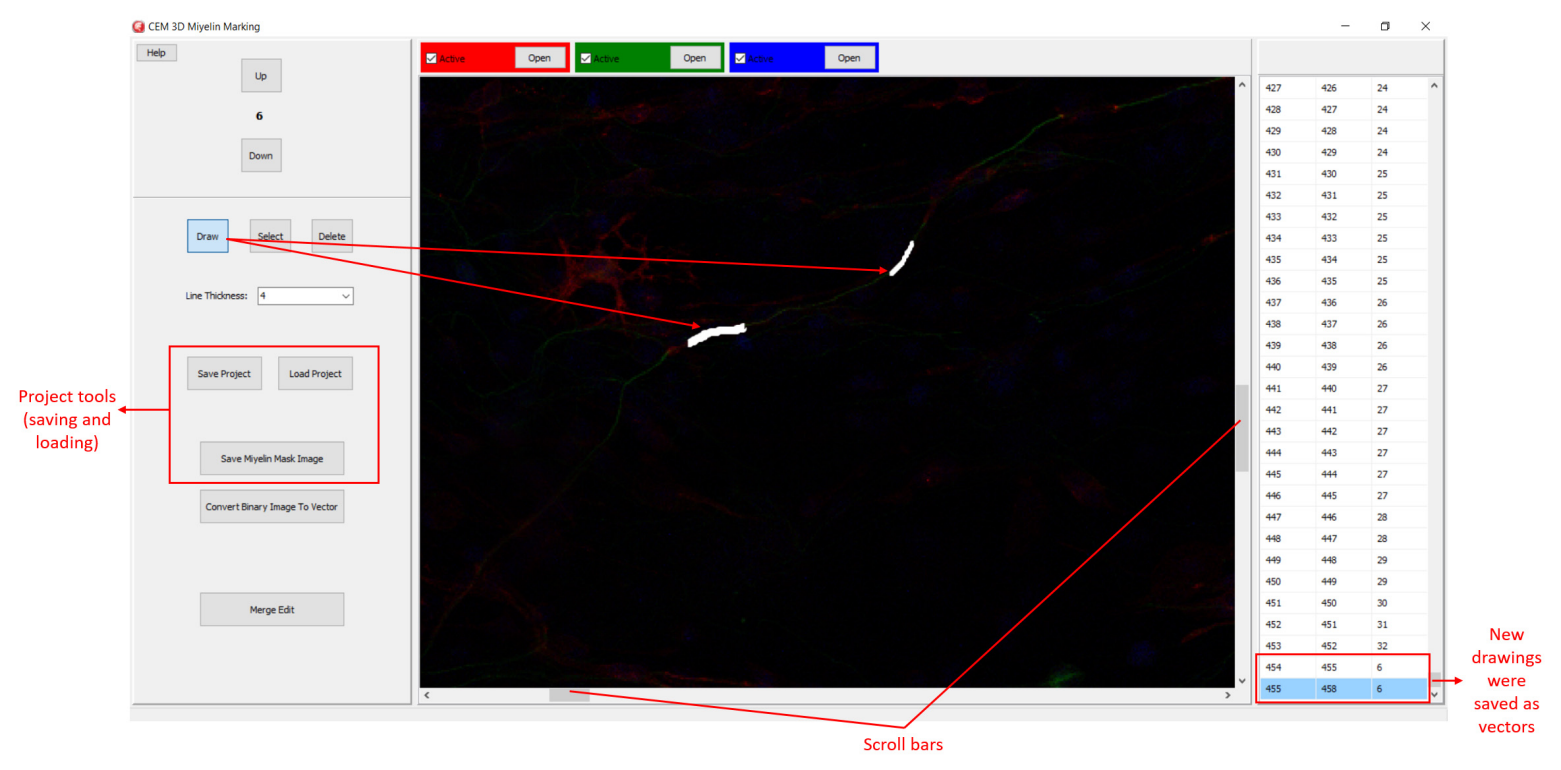
Myelin drawing and saving in CEM3D. The relevant buttons and myelin vectors are marked.

In CEM3D, a new project is started by loading oligodendrocyte, axon, and nucleus images, red, green, and blue channels respectively in the example ( Figure 2). Optionally, candidate myelin image, which is converted to vectors using the included module (see below), is loaded. Users can save and reopen projects. In CEM3D, users can zoom using the mouse wheel and can move in the x-y axes and z-axis using scroll bars and buttons respectively ( Figure 2 and Figure 3).

**Figure 4.**
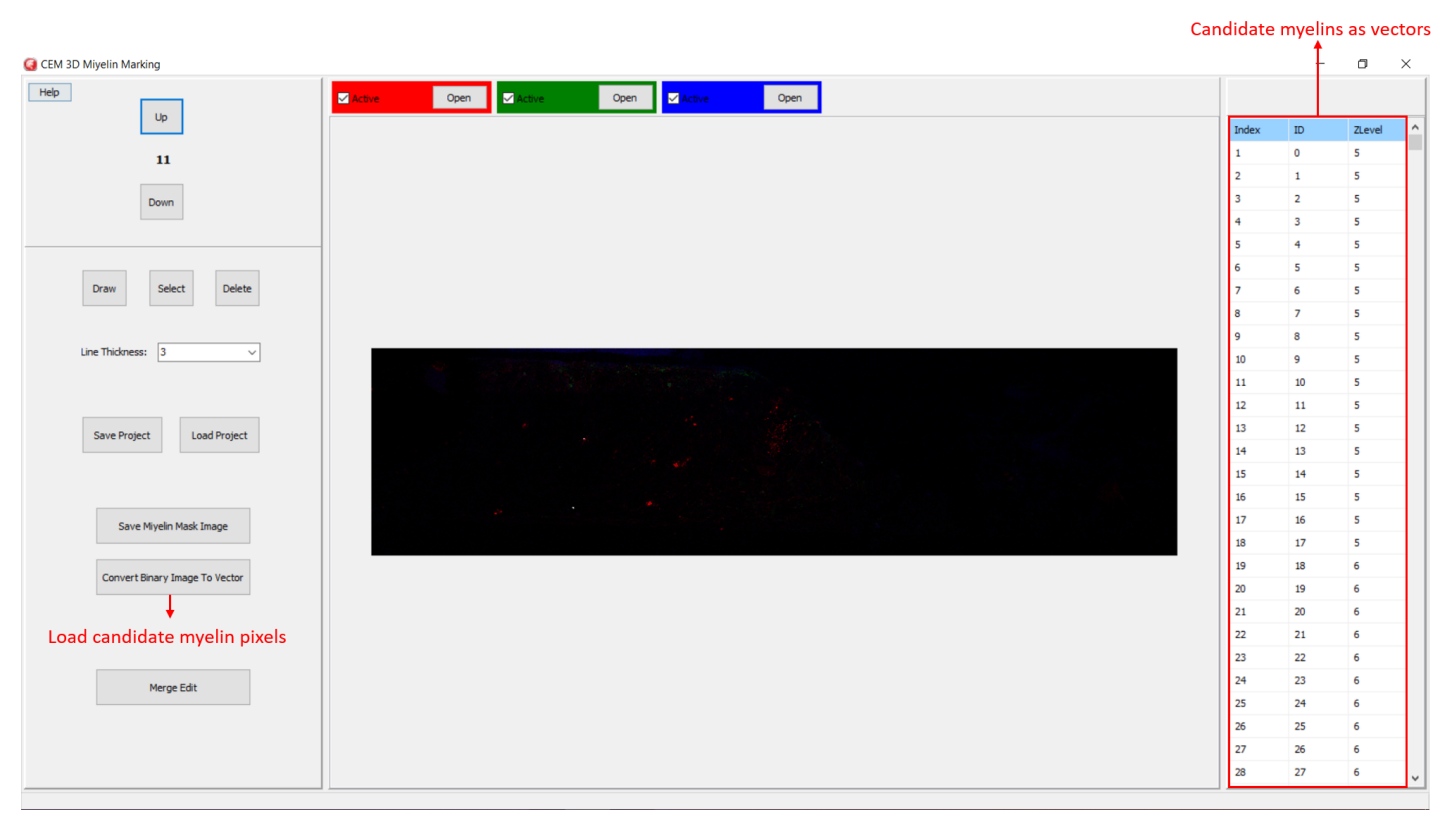
Loading CEM output image. To load candidate myelin pixels, use “Convert Binary Image to Vector” button.

Myelin pixels may be marked at various thickness values ( Figure 3). CEM3D records myelin drawings as vectors in the “.iev” files. These vectors can be modified or deleted in CEM3D ( Figure 3). Optionally, to facilitate myelin detection, the candidate myelins, can be loaded from CEM. Myelin identification using CEM is described in detail in 5. Output of CEM is a binary image, which is converted to vectors using the included module ( Figure 4). Note that the conversion will overwrite your existing myelin vectors.

**Figure 5.**
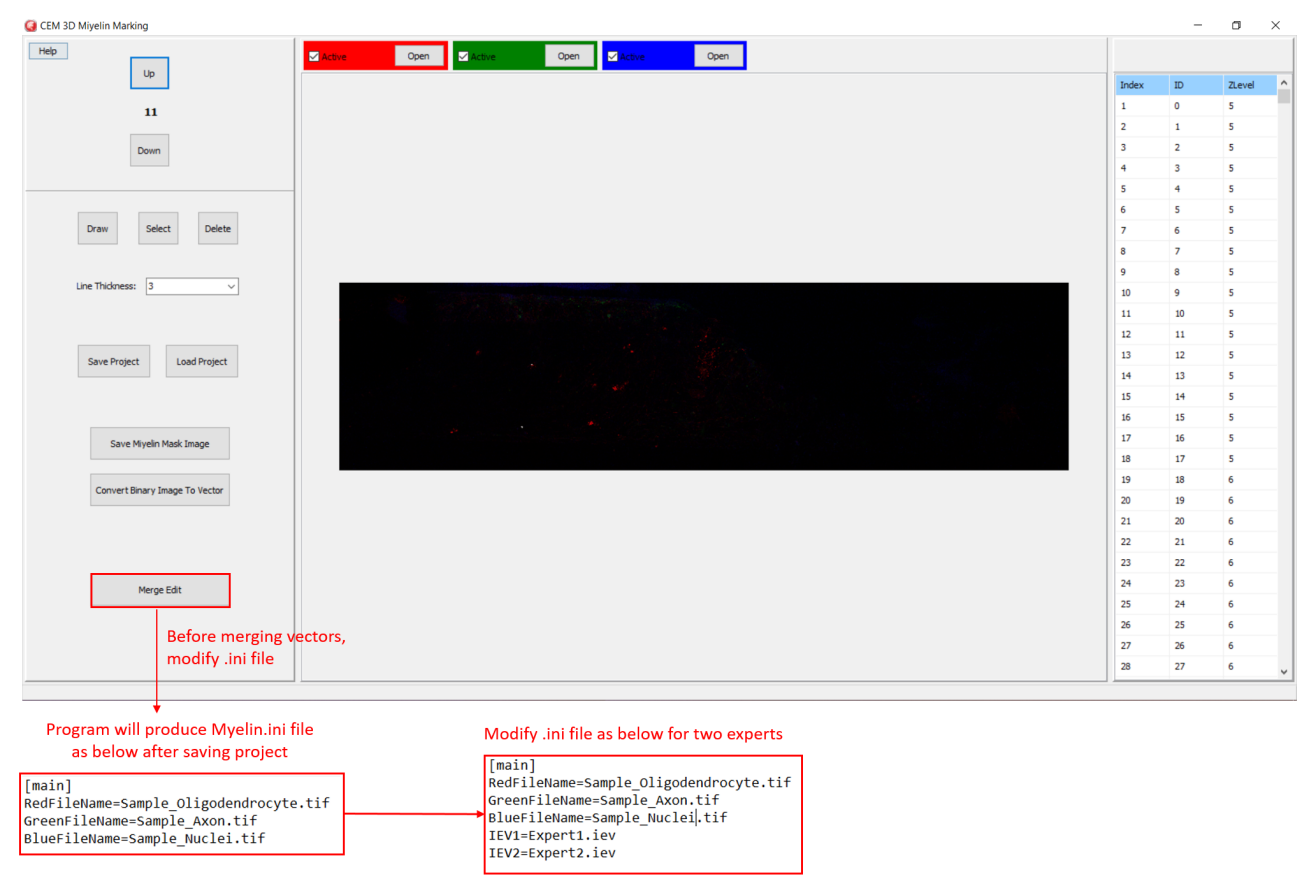
Visualizing two myelin vectors simultaneously. Modify .ini file as in the lower panels and load it using “Merge Edit” button.

**Figure 6.**
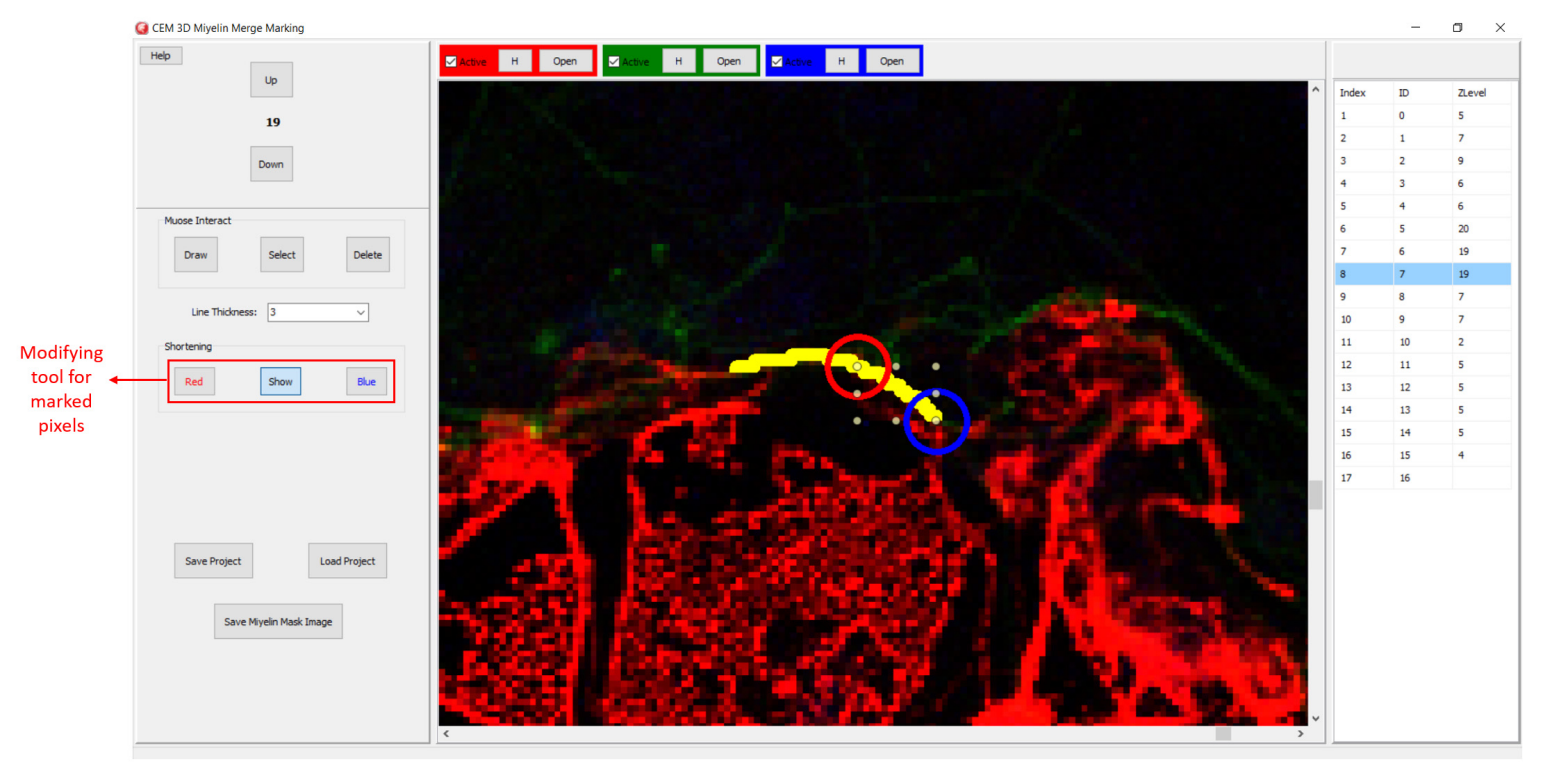
Modifying the myelin vectors. CEM candidate myelins or two experts’ markings can be shortened, deleted or drawn over.

Additionally, CEM candidate myelins or two experts’ myelin vectors can be visualized. First, rename and copy the .iev file containing second myelin vectors to the same folder. Next, modify the .ini files as shown in Figure 5. After loading the modified .ini file using ‘Merge Edit’ button, myelin vectors will be shown in two different colors ( Figure 6). These vectors can be modified as in Figure 6.

Once done with marking, users can convert the myelin vectors into an image using the “Save Myelin Mask Image” button. We implemented this strategy to extract gold standard myelin ground truths.

### Comparative analysis

The myelin marked by two experts were compared against the gold standards. Experts’ precision for each image was calculated as described in 8. The average precision was calculated as mean of precision values of each expert for each image.

### Operation

CEM3D is written in Pascal with the Delphi XE5 platform. The program can be run on 64-bit Microsoft Windows operating systems.

## Results

In this study, myelin was marked by two experts on previously acquired oligodendrocyte and neuron co-culture images ^5^ using the described workflow (see *Implementation*). A third expert evaluated their markings and extracted gold standard myelin ground truths. The ground truth images were saved as TIF on CEM3D ^6^. All images are available (see below).

Because each image covered a large volume (approximately 2 × 8 mm by 30–50 μm), the entire process took approximately five work days. We estimated that it would have taken several weeks using conventional methods. Thus, CEM3D enabled collaboration of three experts for accelerated myelin ground truth extraction.

Next, we calculated experts’ performance. When compared to the gold standards that we extracted, two experts averaged 48.39% precision. The highest precision of an expert was 87.95% for one image. In comparison, our customized-CNN and Boosted Trees consistently reached precision values over 99% ^8^. These results suggest that, machine learning methods can outperform human annotators once trained with accurately labeled data.

## Conclusion

CEM3D ^6^ accelerates annotation of multi-spectral images. As an example, we used it to annotate myelin, which can only be identified as co-localization of neuron and oligodendrocyte membranes within certain criteria. CEM3D’s visualization features simplified inter-expert collaboration and validation. Moreover, myelin ground truths accompanying this manuscript are a resource for the researchers working on segmenting myelin as well as other features in multi-spectral images.

## Data availability

### Underlying data

Image Data Resource: A Multi-Spectral Myelin Annotation Tool for Machine Learning Based Myelin Quantification. Project number idr0100; https://doi.org/10.17867/10000152 ^10^.

This project contains the raw image files analyzed in this article.

Data are available under the terms of the Creative Commons Attribution 4.0 International license (CC-BY 4.0).

## Software availability

**CEM and CEM3D are available from:** https://github.com/ArgenitTech/Neubias.

**Archived source code as at the time of publication:** https://doi.org/10.5281/zenodo.4108321 ^6^.

**License:** Non-Profit Open Software License 3.0 (NPOSL-3.0).
