# A multi-spectral myelin annotation tool for machine learning based myelin quantification

**DOI:** 10.12688/f1000research.27139.2

**Published:** 2022-03-09

**Authors:** Abdulkerim Çapar, Sibel Çimen, Zeynep Aladağ, Dursun Ali Ekinci, Umut Engin Ayten, Bilal Ersen Kerman, Behçet Uğur Töreyin

**Affiliations:** 1 Informatics Institute, Istanbul Technical University, Istanbul, 34469, Turkey; 2 Argenit Akıllı Bilgi Teknolojileri, Istanbul, 34469, Turkey; 3 Department of Electronics and Communication Engineering, Yildiz Technical University, Istanbul, 34220, Turkey; 4 Regenerative and Restorative Medicine Research Center, Istanbul Medipol University, Istanbul, 34810, Turkey; 5 School of Medicine Department of Histology and Embryology, Istanbul Medipol University, Istanbul, 34810, Turkey

**Keywords:** myelin annotation tool, myelin quantification, fluorescence images, machine learning, image analysis

## Abstract

Myelin is an essential component of the nervous system and myelin damage causes demyelination diseases. Myelin is a sheet of oligodendrocyte membrane wrapped around the neuronal axon. In the fluorescent images, experts manually identify myelin by co-localization of oligodendrocyte and axonal membranes that fit certain shape and size criteria. Because myelin wriggles along x-y-z axes, machine learning is ideal for its segmentation. However, machine-learning methods, especially convolutional neural networks (CNNs), require a high number of annotated images, which necessitate expert labor. To facilitate myelin annotation, we developed a workflow and software for myelin ground truth extraction from multi-spectral fluorescent images. Additionally, to the best of our knowledge, for the first time, a set of annotated myelin ground truths for machine learning applications were shared with the community.

## Introduction

Myelin degeneration causes neurodegenerative disorders, such as multiple sclerosis (MS) ^1, 2^. There are no remyelinating drugs. Myelin quantification is essential for drug discovery, which often involves screening thousands of compounds ^3^. Currently, myelin quantification is manual, and labor-intensive. Automation of quantification using machine learning can facilitate drug discovery by reducing time and labor costs ^4^. However, myelin annotation suffers the same limitations as manual quantification. To assist researchers and bioimage analysts, we developed a workflow and software for myelin ground truth extraction from multi-spectral fluorescent images.

**Figure 1.**
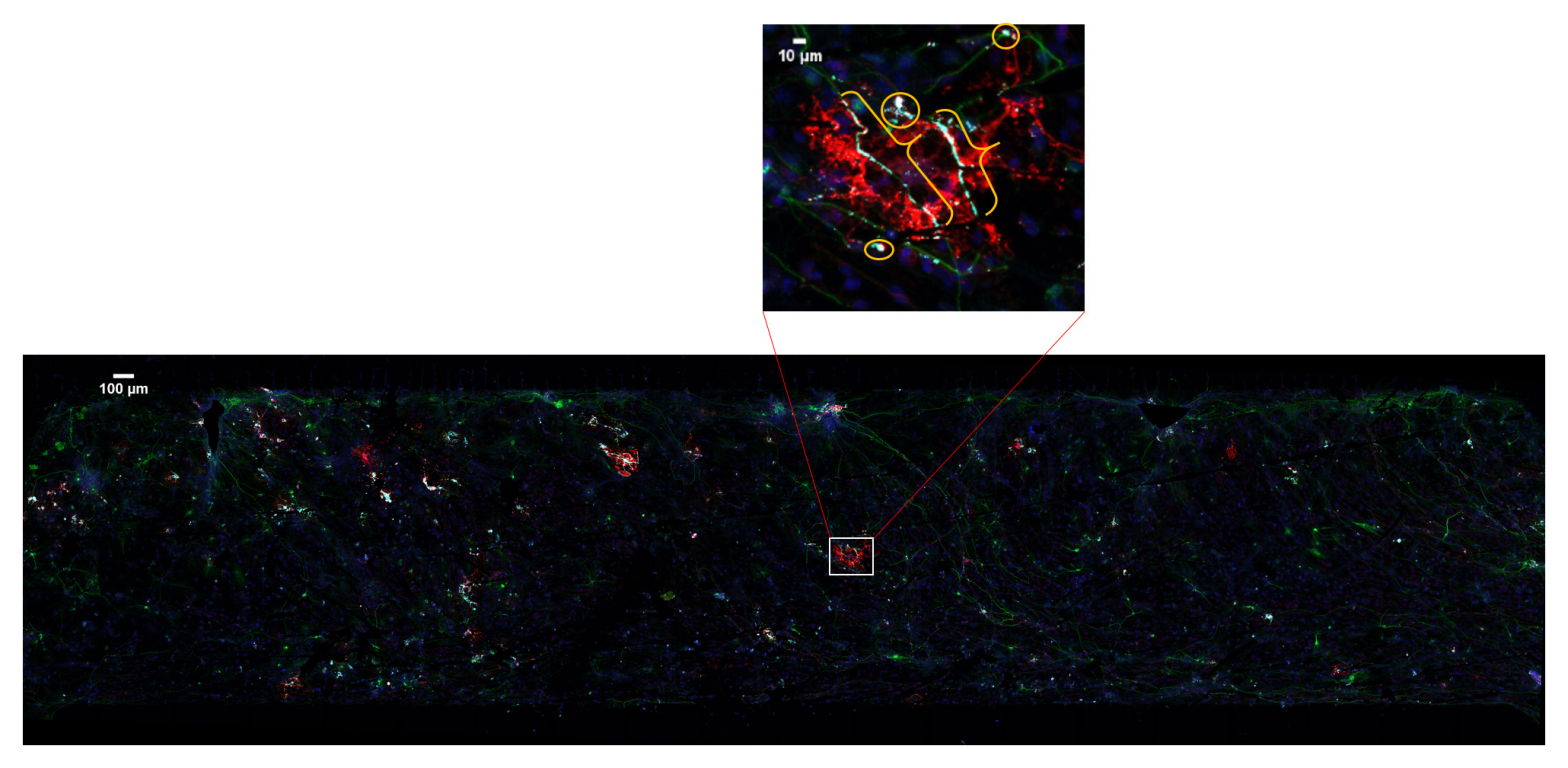
An example of multi-spectral fluorescent image. 20× confocal microscopy image tiles were stitched together covering approximately 2 × 8 mm by 30–50 μm volume. Boxed area is enlarged to show myelin (brackets) and the false positive pixels (circles).

Myelin is formed by oligodendrocytes wrapping the axons ^5^. It is identified by continuous co-localization of cellular extensions that span multiple channels and z-sections ( Figure 1). In our workflow, co-localizing pixels, candidate myelins, were determined using Computer-assisted Evaluation of Myelin (CEM) software that we previously developed ^6^. In this context, CEM software functions as a candidate myelin detection program because it simply identifies overlapping pixels. Briefly, CEM removes cell bodies, defined as the overlap of nuclei and cellular marker, and identifies overlapping pixels between remaining oligodendrocyte and neuron channels ^6^.

In the current study, the CEMotate tool ^7^ was developed to efficiently evaluate these candidate myelins and to extract myelin ground truths. Using CEMotate, an RGB-composite z-section image, corresponding CEM output image, and expert’s markings can be visualized simultaneously to decide whether to keep or remove candidate pixels (see *Implementation*). The user can move along x-y-z axes and show/hide channels, images, and markings. Markings from the -1/+1 z-sections can be viewed simultaneously. Finally, CEMotate allows simultaneous visualization of myelin markings of two experts, which is important for inter-expert comparison.

Using the described workflow, we annotated five images encompassing approximately 2 × 8 mm by 30–50 μm volume. The entire process, which would have taken several weeks, took approximately 5 days. More than 30,000 feature images were extracted from these five images and were used for testing various machine-learning methods ^8– 10^. The annotated images, which are available with the manuscript, are a resource for the researchers working not only on myelin detection but also on segmenting multi-spectral images.

## Methods

### Image acquisition

Images were previously acquired ^6^. Briefly, co-cultures of mouse embryonic stem cell-derived oligodendrocytes and neurons were grown in microfluidic chambers. After myelin formation, cells were fixed in paraformaldehyde and were stained with 1:1,000 mouse or rabbit anti-TUJ1 (Covance), 1:50 rat anti-MBP (Serotec), and DAPI (Sigma). Images were acquired on Zeiss confocal microscopes as tiles approximately 2mm×8mm. The z-axis, 30–50 µm, was covered by 1-µm-thick optical z-sections. The tiles were stitched together on Zen software (Zeiss). No further processing was done.

### Implementation

**Figure 2.**
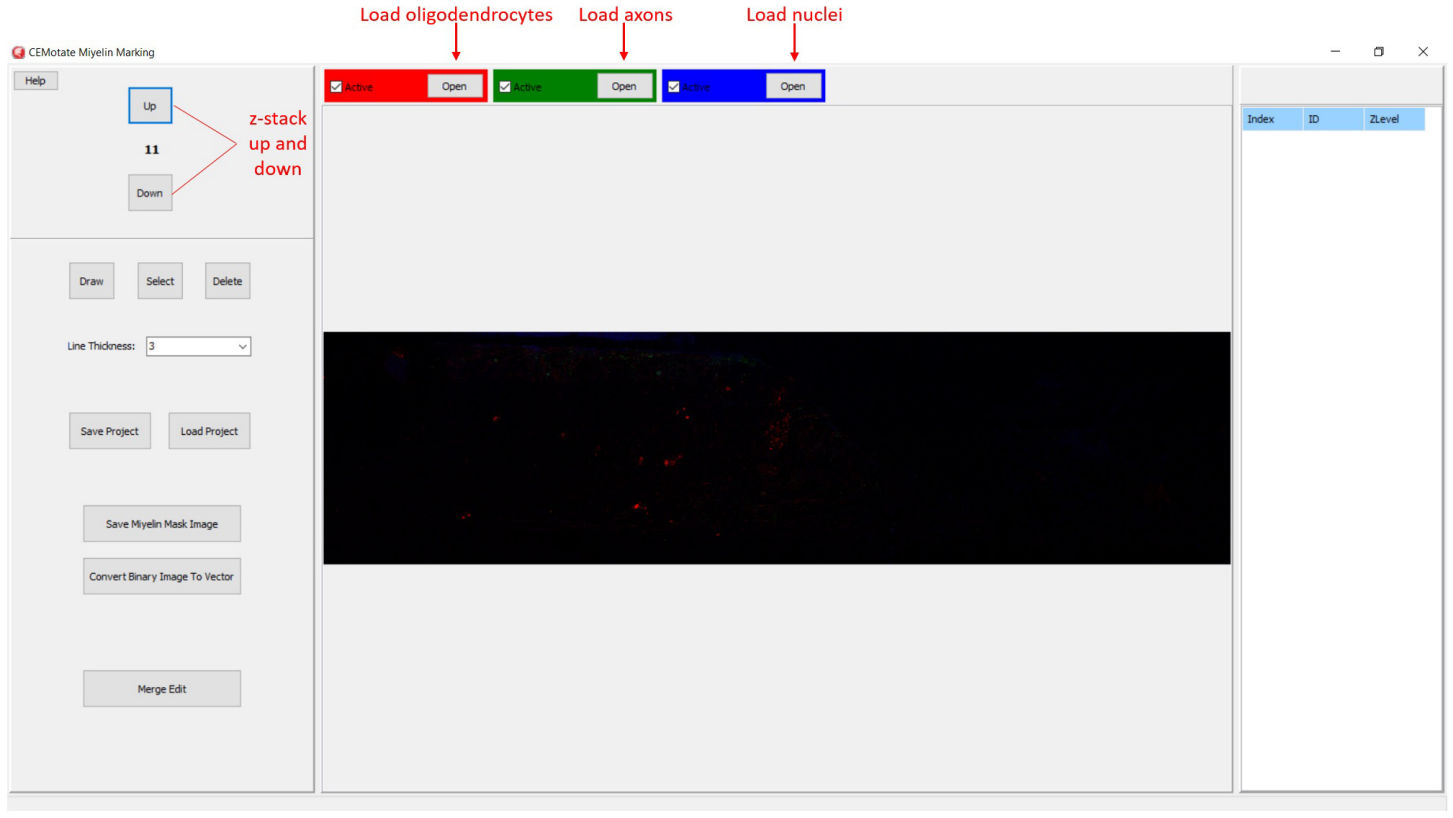
Starting a new project in CEMotate. Buttons for loading oligodendrocyte, axon, and nucleus images, and navigating the z-stack button to up and down are marked.

**Figure 3.**
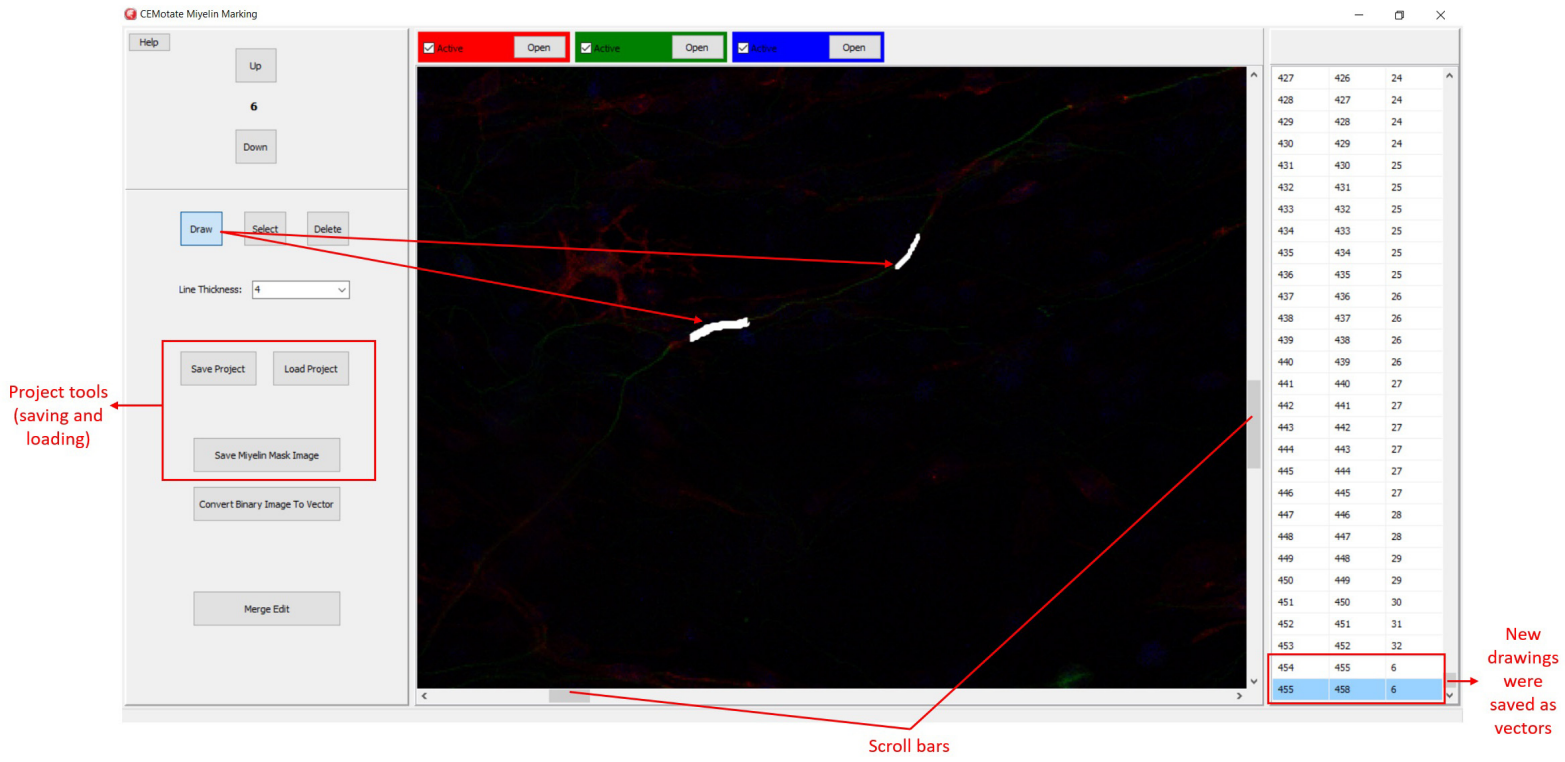
Myelin drawing and saving in CEMotate. The relevant buttons and myelin vectors are marked.

In CEMotate, a new project is started by loading oligodendrocyte, axon, and nucleus images, red, green, and blue channels respectively in the example ( Figure 2). Users can save and reopen projects. In CEMotate, users can zoom using the mouse wheel and can move in the x-y axes and z-axis using scroll bars and buttons respectively ( Figure 2 and Figure 3).

**Figure 4.**
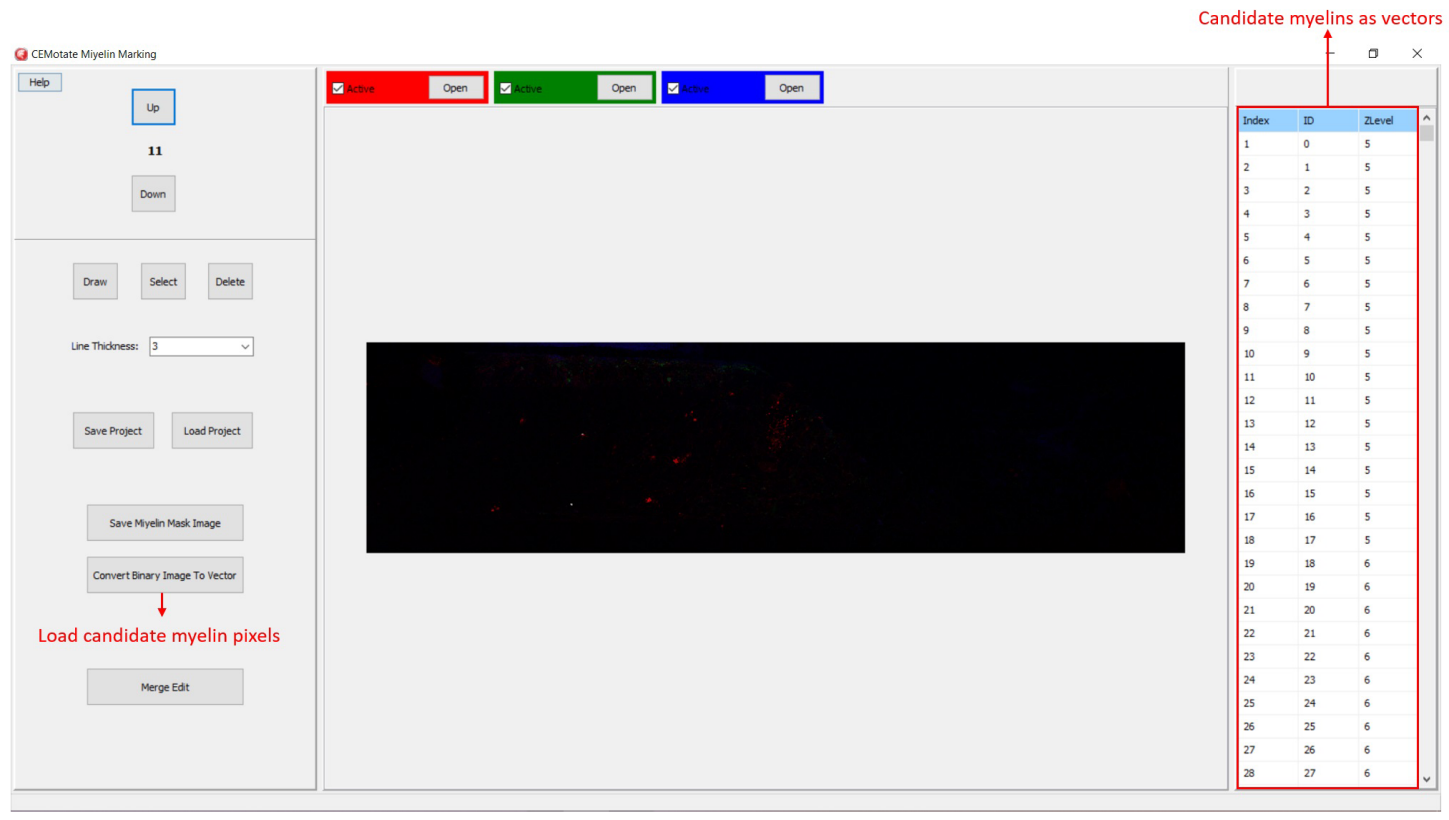
Loading CEM output image. To load candidate myelin pixels, use “Convert Binary Image to Vector” button.

Myelin pixels may be marked at various thickness values ( Figure 3). CEMotate records myelin drawings as vectors in the “.iev” files. These vectors can be modified or deleted in CEMotate ( Figure 3). Optionally, to facilitate myelin detection, the candidate myelins can be loaded from CEM ^6^ or another source that generates binary images of myelin markings. Myelin identification using CEM is described in detail in 6. Output of CEM, is a binary image, which is converted to vectors using the included module ( Figure 4). Note that the conversion will overwrite your existing myelin vectors.

**Figure 5.**
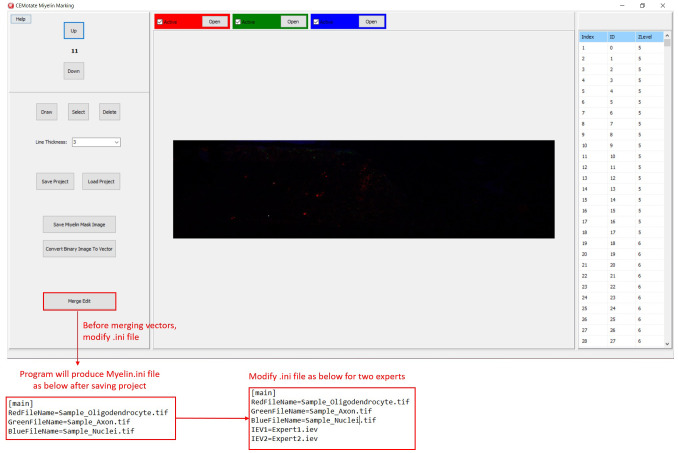
Visualizing two myelin vectors simultaneously. Modify .ini file as in the lower panels and load it using “Merge Edit” button.

**Figure 6.**
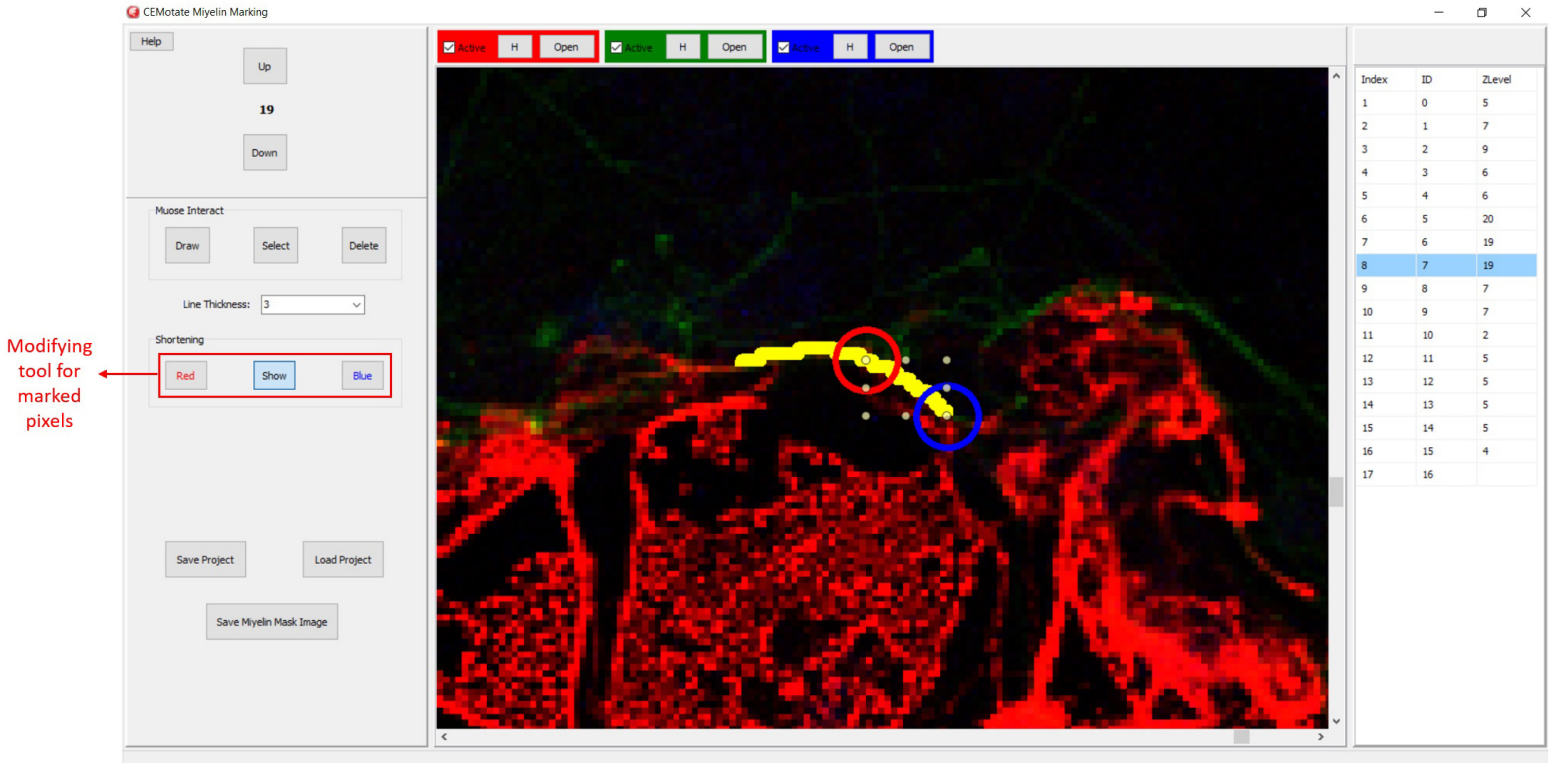
Modifying the myelin vectors. CEM candidate myelins or two experts’ markings can be shortened, deleted or drawn over.

Additionally, myelin regions from two sources can be visualized simultaneously. This allows visualization of myelins annotated by experts and CEM, to do so, first, rename and copy the ‘‘.iev’’ file containing second myelin vectors to the same folder. Next, modify the ‘‘.ini” files as shown in Figure 5. After loading the modified ‘‘.ini” file using the ‘Merge Edit’ button, myelin vectors will be shown in two different colors ( Figure 6). These vectors can be modified as in Figure 6.

Once done with marking, users can convert the myelin vectors into an image using the “Save Myelin Mask Image” button. We implemented this strategy to extract gold standard myelin ground truths.

### Comparative analysis

The myelins marked by two experts were compared against the gold standards. Experts’ precision for each image was calculated as described in 9. The average precision was calculated as mean of precision values of each expert for each image.

### Operation

CEMotate is written in Pascal with the Delphi XE5 platform. The program can be run on 64-bit Microsoft Windows operating systems.

## Results

In this study, myelin was marked by two experts on previously acquired oligodendrocyte and neuron co-culture images ^6^ using the described workflow (see *Implementation*). A third expert evaluated their markings and extracted gold standard myelin ground truths. The ground truth images were saved as TIF on CEMotate ^7^. All images are available (see below).

Each image covered a large volume (approximately 2 × 8 mm by 30–50 μm).

While CEM determined the candidate myelins on five images in approximately 43 minutes, ML approach took only 1.04 seconds for the same process ^8^ ( Table 1). Extracting the gold standard myelin ground truths from five images with candidate pixels that were determined by CEM took approximately another 35 hours for one expert. This process involved determining FPs and FNs on ImageJ. The same process took approximately 20 hours for an expert using CEMotate. Thus, over 40% of time was saved ( Table 2). Moreover, CEMotate enabled collaboration of three experts for accelerated myelin ground truth extraction. Because ImageJ does not have such a feature, we could not directly compare the times saved for this process.

**Table 1.** Time comparison to detect myelin in five images for CEM and ML Approach.

	*CEM*	*ML Approach ^9^*
**Time (~)**	43 min	1.04 sec

**Table 2.** Time comparison for ImageJ and CEMotate annotation.

	*ImageJ*	*CEMotate*
**Time (~)**	35 hours	20 hours

**Table 3.** Experts’ average precisions on candidate myelin pixels of five images.

	*Expert 1*	*Expert 2*
**Average Precisions**	36.23%	60.54%

CEM identified 219032 candidate myelin pixels on five images. Two experts identified TP myelins. A third expert evaluated these results to obtain the gold standard myelin ground truths which covered 9550 pixels. To the best of our knowledge, this is the first time myelin ground truths are shared with the science community.

Next, we calculated each expert's performance ( Table 3). Two experts averaged 48.39% precision. The highest precision of an expert was 87.95% for one image. In comparison, our customized-CNN and Boosted Trees consistently reached precision values over 99% ^8^. These results suggest that, machine learning methods can outperform human annotators once trained with accurately labeled data.

## Conclusion

CEMotate ^7^ accelerates annotation of multi-spectral images. As an example, we used it to annotate myelin, which can only be identified as co-localization of neuron and oligodendrocyte membranes within certain criteria. CEMotate’s visualization features simplified inter-expert collaboration and validation. Moreover, myelin ground truths accompanying this manuscript are a resource for the researchers working on segmenting myelin and other features in multi-spectral images.

## Data availability

### Underlying data

Image Data Resource: A Multi-Spectral Myelin Annotation Tool for Machine Learning Based Myelin Quantification. Project number idr0100; https://doi.org/10.17867/10000152 ^11^.

This project contains the raw image files analyzed in this article.

Data are available under the terms of the Creative Commons Attribution 4.0 International license (CC-BY 4.0).

## Software availability

**CEM and CEMotate are available from:** https://github.com/ArgenitTech/Neubias.

**Archived source code as at the time of publication:** https://doi.org/10.5281/zenodo.4108321 ^7^.

**License:** Non-Profit Open Software License 3.0 (NPOSL-3.0).
