# Supplementary material for: A multi-spectral myelin annotation tool for machine learning based myelin quantification
Source: F1000Res. 2023 Nov 15;9:1492. Originally published 2020 Dec 21. [Version 4] doi: 10.12688/f1000research.27139.4 (PMC10660289; doi:10.12688/f1000research.27139.4)
Supplement: Peer review history

# Reviewer report: Reviewer response for version 4

**DOI:** 10.5256/f1000research.158759.r223202

**Published:** 2023-11-20

**Authors:** Mustafa Ozuysal

**Affiliations:** 1 Department of Computer Engineering, Izmir Institute of Technology, Urla, Turkey

The revision addresses all my previous concerns.

Are the conclusions about the tool and its performance adequately supported by the findings presented in the article?

Yes

Is the rationale for developing the new software tool clearly explained?

Yes

Is the description of the software tool technically sound?

Yes

Are sufficient details of the code, methods and analysis (if applicable) provided to allow replication of the software development and its use by others?

Partly

Is sufficient information provided to allow interpretation of the expected output datasets and any results generated using the tool?

Partly

Reviewer Expertise:

Computer vision, image classification, object detection and tracking

I confirm that I have read this submission and believe that I have an appropriate level of expertise to confirm that it is of an acceptable scientific standard.

# Reviewer report: Reviewer response for version 3

**DOI:** 10.5256/f1000research.133487.r154267

**Published:** 2022-11-09

**Authors:** Mustafa Ozuysal

**Affiliations:** 1 Department of Computer Engineering, Izmir Institute of Technology, Urla, Turkey

The manuscript describes an annotation tool for myelin sheets in stacks of fluorescent images and a novel data set annotated using this tool. Annotation performance using the proposed tool is compared to existing software.

 Overall, the article is clearly written and the tool is well motivated. The ability for experts to work collaboratively on the same data is an important aspect of the new tool that might both accelerate annotation and improve the quality of the annotations.

 I think the manuscript could be improved in the following directions to be more informative:

- The results section mentions three experts without qualifying their expertise level. I think it would be better to include their area and level of expertise to allow the reader better interpret the tabulated results.
- The training data used to the custom-CNN/Boosted Tree approach is not immediately clear. Is it trained using the data annotated with the proposed tool or with CEM? The reference [8] indicates the latter but I think it would be good to mention this explicitly.
- The collaborative editing aspect could be expanded if it allows interaction between experts beyond simple parallelism. The level of collaboration allowed by the tool is not clear from the text.

Few minor comments:

- Second paragraph in the Results section (singe sentence) is redundant since the Methods section already provides the same information.
- Some sentences in the Implementation section are phrased in the tone of a user's manual. They should be rephrased to be consistent with the style of the article. "conversion will overwrite *your* existing myelin vectors" -> "conversion overwrites existing myelin vectors"

Are the conclusions about the tool and its performance adequately supported by the findings presented in the article?

Yes

Is the rationale for developing the new software tool clearly explained?

Yes

Is the description of the software tool technically sound?

Yes

Are sufficient details of the code, methods and analysis (if applicable) provided to allow replication of the software development and its use by others?

Partly

Is sufficient information provided to allow interpretation of the expected output datasets and any results generated using the tool?

Partly

Reviewer Expertise:

Computer vision, image classification, object detection and tracking

I confirm that I have read this submission and believe that I have an appropriate level of expertise to confirm that it is of an acceptable scientific standard, however I have significant reservations, as outlined above.

# Reviewer report: Reviewer response for version 3

**DOI:** 10.5256/f1000research.133487.r136045

**Published:** 2022-04-27

**Authors:** Predrag Janjic

**Affiliations:** 1 Research Center for Computer Science and Information Technology, Macedonian Academy of Sciences and Arts, Skopje, North Macedonia

It is acknowledged that the authors accepted the non critical suggestions.

Are the conclusions about the tool and its performance adequately supported by the findings presented in the article?

Partly

Is the rationale for developing the new software tool clearly explained?

Yes

Is the description of the software tool technically sound?

No

Are sufficient details of the code, methods and analysis (if applicable) provided to allow replication of the software development and its use by others?

No

Is sufficient information provided to allow interpretation of the expected output datasets and any results generated using the tool?

Partly

Reviewer Expertise:

Computational neuroscience, structural studies of white matter, dynamical models of glial membrane.

I confirm that I have read this submission and believe that I have an appropriate level of expertise to confirm that it is of an acceptable scientific standard.

# Reviewer report: Reviewer response for version 2

**DOI:** 10.5256/f1000research.121217.r126756

**Published:** 2022-04-08

**Authors:** Predrag Janjic

**Affiliations:** 1 Research Center for Computer Science and Information Technology, Macedonian Academy of Sciences and Arts, Skopje, North Macedonia

The authors have addressed the issues within the initial review carefully.

 I would suggest that the introduction stresses clearly that the specific nature of myelin identification and annotation difficulties this work addresses matter specifically for fluorescent imaging due to the particular nature of the stain and the imaging itself. The legacy EM studies do not meet the same problems necessarily, principally the granular appearance of myelin due to the nature of localization of the fluorophore. The granularity of myelin in this imaging is the critical feature making the annotation of these images pixel/particle based and very laborious.

 The concluding statement in the following paragraph (bold), appearing in the Abstract as well:

 " *CEM identified 219032 candidate myelin pixels on five images. Two experts identified TP myelins. A third expert evaluated these results to obtain the gold standard myelin ground truths which covered 9550 pixels.* ***To the best of our knowledge, this is the first time myelin ground truths are shared with the science community.*"**

 I believe this is correct just **for fluorescent imaging**, since annotated EM images have been shared, at least within data archives and along qualified requests after the availability has been stated in the papers.

 Will appreciate if authors would fix this at both places.

Are the conclusions about the tool and its performance adequately supported by the findings presented in the article?

Partly

Is the rationale for developing the new software tool clearly explained?

Yes

Is the description of the software tool technically sound?

No

Are sufficient details of the code, methods and analysis (if applicable) provided to allow replication of the software development and its use by others?

No

Is sufficient information provided to allow interpretation of the expected output datasets and any results generated using the tool?

Partly

Reviewer Expertise:

Computational neuroscience, structural studies of white matter, dynamical models of glial membrane.

I confirm that I have read this submission and believe that I have an appropriate level of expertise to confirm that it is of an acceptable scientific standard.

# Response

**Published:** 2022-04-24

**Authors:** Bilal Kerman

**Affiliations:** Istanbul Medipol University, Turkey

Dear Dr. Janjic,

 Thank you very much for your approval and again noticing an important detail. We updated the manuscript to emphasize that this tool is for fluorescent images. Analysis of electron microscopy images of myelin present a different set of challenges.

# Reviewer report: Reviewer response for version 1

**DOI:** 10.5256/f1000research.29981.r76521

**Published:** 2021-02-08

**Authors:** Predrag Janjic

**Affiliations:** 1 Research Center for Computer Science and Information Technology, Macedonian Academy of Sciences and Arts, Skopje, North Macedonia

The manuscript introduces a 3D extension of myelin annotation tool reported in Ref[5], now applicable to fluorescent stacks. Although the main rationale to develop an automated tool for producing ground truth images is clear, and the importance has been laid out, methodological details and comparative data are missing for a researcher dealing with myelin imaging to get an idea of what the tool is really computationally doing in order to decide on its practical utility.

 Please rework the Introduction and despite rather harsh length constraints add some minimal description of what algorithms in Ref[5] do.

**Image acquisition:**

- Extend on the image and image processing details like the size of the captures, pixel size, deconvolving or not, depth corrections (you have some of it in the Results).

**Implementation:**

- Please extend on what has been done to integrate CEM tool, and parallel visualization of myelin in subsequent planes, figures Fig.5 and Fig.6., elaborating the utility of this step which is the main procedural added value of the presented tool. Please move to additional material or remove Fig.1 - Fig.4., which are more of a user guide and are disruptive in the value presentation.

**Comparative analysis:**

- Some more data is needed, new or from Ref[8] for a reader to be able to get the overall impression. Please consider adding a *Benchmarking* paragraph where you would extend a bit on some benchmarking given within the Results, with an estimate (table) of the computational time needed for the CEM scope per slice, and the total time to process a whole stack, all in order a potential end user to get an impression of the effort needed. 

**Results:**

- Please extend on limitations & issues of the CEM3D, and try to estimate if possible specific performance over a whole stack using this extension. (which is the actual improvement and a gain compared to relying only on CEM).  

Are the conclusions about the tool and its performance adequately supported by the findings presented in the article?

Partly

Is the rationale for developing the new software tool clearly explained?

Yes

Is the description of the software tool technically sound?

No

Are sufficient details of the code, methods and analysis (if applicable) provided to allow replication of the software development and its use by others?

No

Is sufficient information provided to allow interpretation of the expected output datasets and any results generated using the tool?

Partly

Reviewer Expertise:

computational neuroscience, structural studies of white matter, dynamical models of glial membrane.

I confirm that I have read this submission and believe that I have an appropriate level of expertise to state that I do not consider it to be of an acceptable scientific standard, for reasons outlined above.

# Response

**Published:** 2022-02-23

**Authors:** Bilal Kerman

**Affiliations:** Istanbul Medipol University, Turkey

We thank Predrag Janjic for his helpful comments. We believe that we address his concerns and the updated manuscript is easier to read and more satisfactory to the readers. Please see our point by point responses below.

 I apologize in the time it took us to update the manuscript. I moved between institutions and it took me a long time to settle in part due to the pandemic restrictions.

**T** **he manuscript introduces a 3D extension of myelin annotation tool reported in Ref[5], now applicable to fluorescent stacks. Although the main rationale to develop an automated tool for producing ground truth images is clear, and the importance has been laid out, methodological details and comparative data are missing for a researcher dealing with myelin imaging to get an idea of what the tool is really computationally doing in order to decide on its practical utility.**

**Please rework the Introduction and despite rather harsh length constraints add some minimal description of what algorithms in Ref[5] do.**

**Response 1: **We updated the Introduction to include: “In this context, CEM software functions as a candidate myelin detection program because it simply identifies overlapping pixels. Briefly, CEM removes cell bodies, defined as the overlap of nuclei and cellular marker, and identifies overlapping pixels between remaining oligodendrocyte and neuron channels ^6^.”

**Image acquisition:**

**Extend on the image and image processing details like the size of the captures, pixel size, deconvolving or not, depth corrections (you have some of it in the Results).**

**Response 2: **The tool described in this publication is not an image processing tool per se. It is main function is annotation of myelin pixels for machine learning studies. Therefore, in this revision, to prevent any confusion, we renamed the tool as CEMotate.

**Implementation:**

**Please extend on what has been done to integrate CEM tool, and parallel   visualization of myelin in subsequent planes, figures Fig.5 and Fig.6., elaborating the utility of this step which is the main procedural added value of the presented tool. Please move to additional material or remove Fig.1 - Fig.4., which are more of a user guide and are disruptive in the value presentation.**

**Response 3: **In this study, the CEM tool is not integrated directly to CEMotate. We used images previously processed by CEM and annotated them using CEMotate. The details of processing by CEM were given in the reference Kerman et al. 2015. The one major goal of this manuscript is to introduce our myelin annotation tool and to describe how to utilize it. Therefore, we believe that Fig. 1 – Fig. 4. are useful for readers.

**Comparative analysis:**

**Some more data is needed, new or from Ref[8] for a reader to be able to get the overall impression. Please consider adding a Benchmarking paragraph where you would extend a bit on some benchmarking given within the Results, with an estimate (table) of the computational time needed for the CEM scope per slice, and the total time to process a whole stack, all in order a potential end user to get an impression of the effort needed.**

**Response 4: The updated text below:**

 We added information on benchmarking and compared the length of the time it takes to use different approaches.

 Results Section – Paragraph 2: Each image covered a large volume (approximately 2 x 8 mm by 30-50 μm). While CEM determined the candidate myelins on five images in approximately 43 minutes, ML approach took only 1.04 seconds for the same process ^8^ (Table 1).  Extracting the gold standard myelin ground truths from five images with candidate pixels that were determined by CEM took approximately another 35 hours for one expert.  This process involved determining FPs and FNs on ImageJ. The same process took approximately 20 hours for an expert using CEMotate. Thus, over 40% of time was saved (Table 2). Moreover, CEMotate enabled collaboration of three experts for accelerated myelin ground truth extraction. Because ImageJ does not have such a feature, we could not directly compare the times saved for this process.

**Table 1. Time comparison to detect myelin in five images for CEM and ML Approach**

***CEM***

***ML Approach ^9^***

**Time (** **~** **)**

 43 min

 1.04 sec

**Table 2. Time comparison for ImageJ and CEMotate annotation**

***ImageJ***

***CEMotate***

**Time (** **~** **)**

 35 hours

 20 hours

 CEM identified 219032 candidate myelin pixels on five images. Two experts identified TP myelins. A third expert evaluated these results to obtain the gold standard myelin ground truths which covered 9550 pixels. To the best of our knowledge, this is the first time myelin ground truths are shared with the science community.

**Table 3. Experts’ average precisions on candidate myelin pixels of five images**

***Expert 1***

***Expert 2***

**Average Precisions**

 36.23%

 60.54%

 Next, we calculated each expert’s performance. Two experts averaged 48.39% precision. The highest precision of an expert was 87.95% for one image. In comparison, our customized-CNN and Boosted Trees consistently reached precision values over 99% ^8^. These results suggest that machine learning methods can outperform human annotators once trained with accurately labeled data.

**Results:**

**Please extend on limitations & issues of the CEM3D, and try to estimate if possible specific performance over a whole stack using this extension. (which is the actual improvement and a gain compared to relying only on CEM).**

  We updated the Results section to include the metrics as described above.
